# A novel cuproptosis-related diagnostic gene signature and differential expression validation in atherosclerosis

**DOI:** 10.1186/s43556-023-00131-5

**Published:** 2023-07-14

**Authors:** Yuting Cui, Yanyu Chen, Ni Gan, Man Li, Wei Liao, Yating Zhou, Qiong Xiang, Xi Gong, Qianqian Guo, Pengwei Hu, Xi-Long Zheng, Desi Shang, Juan Peng, Zhihan Tang

**Affiliations:** 1 Institute of Cardiovascular Disease, Key Laboratory for Arteriosclerology of Hunan Province, Hunan International Scientific and Technological Cooperation Base of Arteriosclerotic Disease, School of Basic Medical Sciences, Hengyang Medical School, University of South China, Hengyang, 421001 China; 2 Hengyang Medical School, The Affiliated Changsha Central Hospital, University of South China, Hengyang, Hunan 421001 China; 3 The Xinjiang Technical Institute of Physics and Chemistry, Chinese Academy of Sciences, Urumqi, 830011 China; 4 Departments of Biochemistry & Molecular Biology and Physiology & Pharmacology, Cumming School of Medicine, University of Calgary, Calgary, AB T2N 4Z6 Canada; 5 Hengyang Medical School, The First Affiliated Hospital, University of South China, Hengyang, Hunan 421001 China

**Keywords:** Atherosclerosis, Atherosclerotic cardiovascular disease, Cuproptosis, Bioinformatics

## Abstract

Atherosclerosis (AS) is a major contributor to morbidity and mortality worldwide. However, the molecular mechanisms and mediator molecules involved remain largely unknown. Copper, which plays an essential role in cardiovascular disease, has been suggested as a potential risk factor. Copper homeostasis is closely related to the occurrence and development of AS. Recently, a new cell death pathway called cuproptosis has been discovered, which is driven by intracellular copper excess. However, no previous studies have reported a relationship between cuproptosis and AS. In this study, we integrated bulk and single-cell sequencing data to screen and identify key cuproptosis-related genes in AS. We used correlation analysis, enrichment analysis, random forest, and other bioinformatics methods to reveal their relationships. Our findings report, for the first time, the involvement of cuproptosis-related genes FDX1, SLC31A1, and GLS in atherogenesis. FDX1 and SLC31A1 were upregulated, while GLS was downregulated in atherosclerotic plaque. Receiver operating characteristic curves demonstrate their potential diagnostic value for AS. Additionally, we confirm that GLS is mainly expressed in vascular smooth muscle cells, and SLC31A1 is mainly localized in macrophages of atherosclerotic lesions in experiments. These findings shed light on the cuproptosis landscape and potential diagnostic biomarkers for AS, providing further evidence about the vital role of cuproptosis in atherosclerosis progression.

**Supplementary Information:**

The online version contains supplementary material available at 10.1186/s43556-023-00131-5.

## Introduction

Over the past 20 years, cardiovascular disease (CVD) has gradually eroded and burdened healthcare systems, and is the leading cause of morbidity and mortality worldwide [1]. AS is the primary cause and pathologic foundation of CVD [2], and the pathophysiological mechanisms responsible for AS are multifactorial, including inflammation, oxidative stress, dysregulated lipid metabolism, and transition metal ion metabolism [3–6]. Despite these promising studies, however, CVD pathogenesis is still poorly understood. Research is needed to identify new pathways and reveal the underlying mechanism in AS that could be targeted therapeutically to reduce the global burden of AS.

Copper is one of the essential trace elements involved in various important biological processes in the human body. In addition, studies show that copper ions are closely related to AS development. Elevated levels of copper ions in the serum and plaques of atherosclerotic patients have been demonstrated [3]. The data from the Giovanni Tarantino group shows that copper bioavailability is also a central factor in early atherosclerosis, as evidenced by the altered serum copper bioavailability predicting early atherosclerosis as the main cardiovascular risk in obese patients with a low prevalence of comorbidities [7]. Excess copper can be deposited in lysosomes, leading to cell death in human umbilical vein endothelial cells (HUVECs). Treatment with the copper ion chelator tetrathiomolybdate (TTM) alleviates this effect [8, 9]. Wei et al. found that the use of copper chelating agents and copper ion carrier inhibitors can reduce the development of atherosclerotic lesions in mice [10], and Völker et al. proposed that copper ions also can induce inflammatory reactions and promote AS-like neointima formation in rats [11]. Moreover, copper ions have also been shown to play important roles in the oxidative modification of low-density lipoprotein (LDL) and vessel wall homeostasis [12, 13]. Therefore, an imbalance in copper homeostasis can lead to the progression of AS [14, 15].

Recently, a new cell death pathway called cuproptosis has been discovered [16]. Cuproptosis relies on mitochondrial respiration and involves the direct binding of copper to the lipoacylated components of the tricarboxylic acid (TCA) cycle, resulting in toxic protein stress and ultimately inducing cell death [16]. Elevated levels of copper ions and copper derivatives are essential for cuproptosis; however, a relationship between cuproptosis and AS has not been reported.

In the present study, we downloaded atherosclerotic disease datasets from GEO, screened out the differential cuproptosis-related genes (DE-CRGs), and investigated the potential mechanisms by bioinformatics analyses. We provided evidence that suggests cuproptosis may be involved in AS, located the potential target genes in vascular smooth muscle cells and macrophages of atherosclerotic lesions, and evaluated their diagnostic value.

## Results

### Identification and correlation analysis of candidate DE-CRGs

**Fig. 1:**
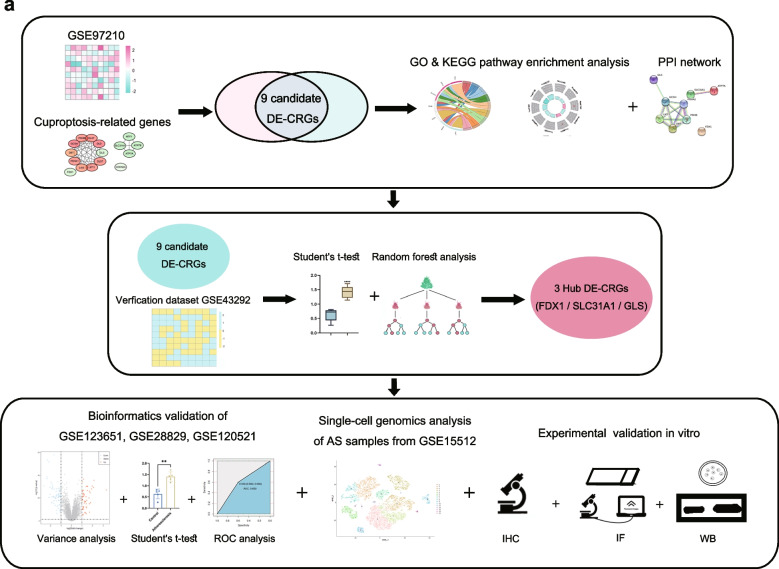
**a** The overall design of this study

**Fig. 2:**
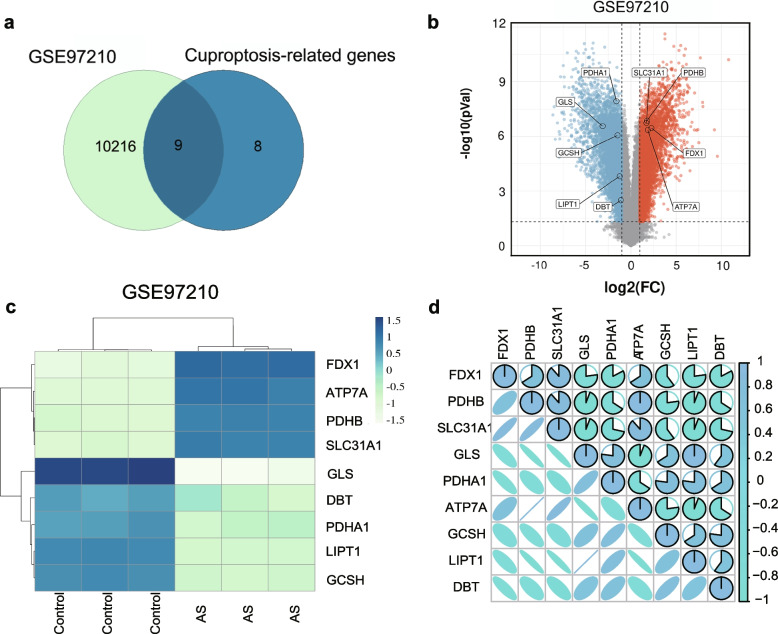
Analysis of differentially expressed genes (DEGs) related to cuproptosis and functional enrichment analysis. **a** Venn diagram showing the cuproptosis-related genes in the GSE97210 datasets. **b** The identified cuproptosis-related DGEs displayed in a volcano plot, upregulated (red) and downregulated (blue). **c** Heat map of the cuproptosis-related genes in the GSE97210 datasets. **d** Correlation analysis of candidate cuproptosis-related genes in GSE97210 datasets

The overall design of this study is displayed in Fig. 1a. To investigate the differential expression of CRGs in atherosclerotic plaques, we curated a catalog of 16 genes (CDKN2A, FDX1, DLD, DLAT, LIAS, GLS, LIPT1, MTF1, PDHA1, PDHB, ATP7A, ATP7B, DBT, GCSH, DLST, and SLC31A1) that function closely with cuproptosis from the published literature [16]. Differential expression analysis (AS vs. normal) identified 10,226 differentially expressed genes (DEGs) with thresholds of |log2FC|≥ 1 and adjusted *p* < 0.05 in GSE97210. By combining the results of 16 CRGs and DEGs in the GSE97210 datasets (normal vs. AS plaque, *n* = 3), we were able to obtain nine DE-CRGs (Fig. 2a). Compared with the normal control, five genes were downregulated, and four genes were upregulated in atherosclerotic plaques, as marked in the volcano plot (Fig. 2b). The expression of the nine DE-CRGs was visualized in a heat map (Fig. 2c). Pearson’s correlation coefficient was used for the analysis, which showed a significant correlation between these nine genes (Fig. 2d). To summarize, AS patients showed significant dysregulation of CRGs in arterial tissues when compared to normal individuals.

### Functional enrichment analysis and PPI network construction with candidate DE-CRGs

**Fig. 3:**
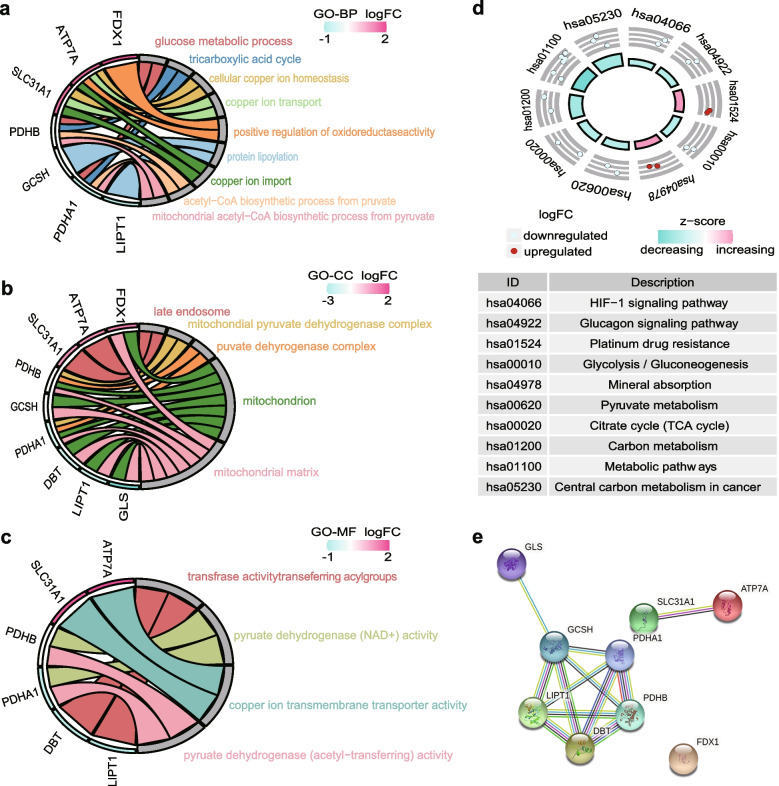
Functional enrichment analysis and protein–protein interaction (PPI) network. **a**-**c** Gene Ontology (GO) enrichment analysis of DEGs of GSE97210 datasets. **a** Biological process (BP); **b** Cellular component (CC); **c** Molecular function (MF). **d** Kyoto Encyclopedia of Genes and Genomes (KEGG) pathway enrichment analysis of DEGs of GSE97210 datasets. **e** Protein–protein-interaction (PPI) network of the cuproptosis-related genes in GSE97210 datasets

Based on GO and KEGG pathway analyses, we explored the potential biological functions and pathways of the nine DE-CRGs. For GO analysis, molecular function (MF) results showed that dehydrogenase, transferase, and copper ion transmembrane transporter activities were significantly demonstrated in the DE-CRGs (Fig. 3a). Biological pathway (BP) analysis indicated enriched primary terms relevant to pyruvate-related enzyme synthesis processes, copper ion import, and protein lipidation (Fig. 3b). In the cellular component (CC) study, the screened DE-CRGs were significantly enriched in the mitochondrial matrix, pyruvate dehydrogenase complex, and late endosome (Fig. 3c). Moreover, investigation of the KEGG pathway analysis primarily suggested that these genes were involved in metabolic pathways, carbon metabolism, and the TCA cycle (Fig. 3d). Subsequently, to explore the interaction between the nine DE-CRGs, the genes were submitted to STING to search for protein–protein interactions and construct a PPI network. The results showed a total of nine nodes and twelve edges, and the proteins were highly connected in the PPI network (Fig. 3e). Taken together, these findings demonstrate that candidate DE-CRGs may play a significant role in the progression of AS.

### Screening of Hub DE-CRGs

**Fig. 4:**
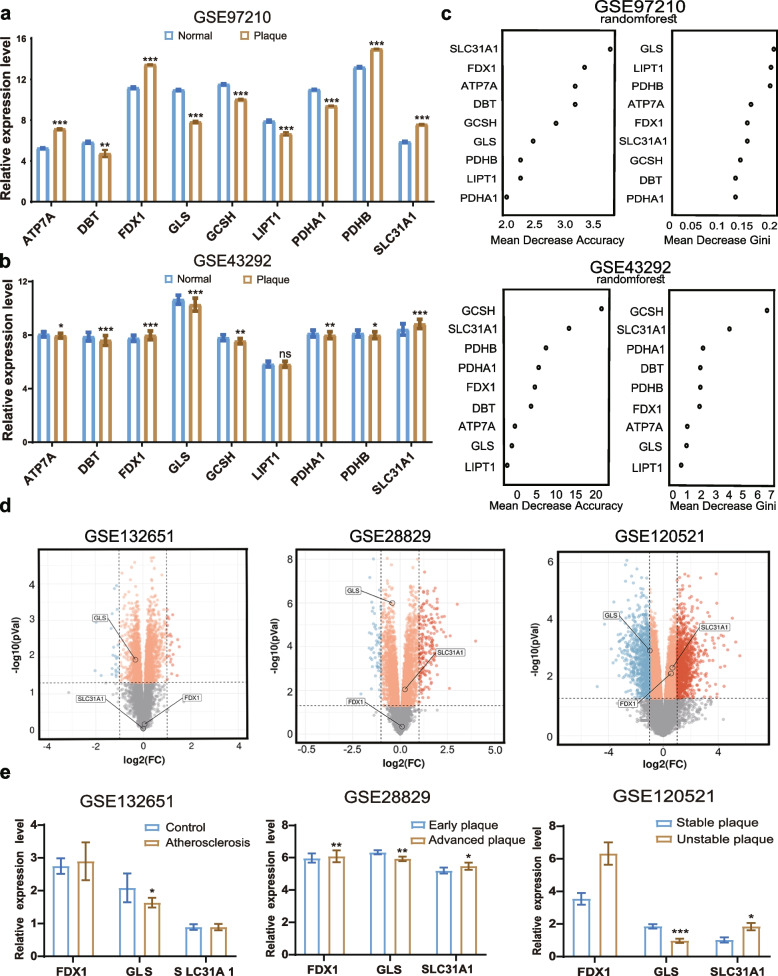
Screening of Hub differentially expressed cuproptosis-related genes (DE-CRGs). **a**-**b** Gene expression levels of nine DE-CRGs were compared in AS (Plaque) and control (Normal) groups with the Student’s t-test in GSE97210 datasets (**a**) and GSE43292 datasets (**b**). Significance markers: ****P < 0.0001, ***P < 0.001, **P < 0.01 and *P < 0.05. **c** Importance ranking of genes in a random forest (RF) model based on datasets GSE43292. **d** Volcano plots displaying the expression of Hub genes in GSE132651 datasets, GSE28829 datasets, and GSE120521 datasets. Upregulated (red) and downregulated (blue) or vice versa. **e** Gene expression level of the three Hub DE-CRGs was compared in normal coronary endothelial (control) and atherosclerotic coronary endothelial cells, early and advanced plaques, and stable and unstable plaques with Student’s t-test in GSE132651 datasets, GSE28829 datasets, and GSE120521 datasets. Significance markers: ****P < 0.0001, ***P < 0.001, **P < 0.01 and *P < 0.05 vs. control group

Given the limitations of single data sources, we introduced another dataset GSE43292 (normal vs. AS plaque, *n* = 32), as a validation set to compare the gene expression levels of the nine DE-CRGs in two datasets. We used the same expression, significance trend (*P* < 0.001), and Random Forest (RF) algorithm to identify the key genes. The results revealed that 4 cuproptosis-related genes, GLS, FDX1, SLC31A1, and GCSH, have the same expression and significance trend (*P* < 0.001) in plaque vs. normal control in the GSE97210 and GSE43292. Among them, FDX1 and SLC31A1 were upregulated, and GLS and GCSH were downregulated (Fig. 4a-b). The RF model was used to conduct deep learning on the sample data of GSE97210 and GSE43292. Considering the score distributions of RF and the gene functions, FDX1, SLC31A1, and GLS were identified as hub genes (Fig. 4c). To further validate the accuracy of our previous analysis, we selected three stages of atherosclerotic datasets: GSE132571 (normal vs. atherosclerotic endothelial, *n* = 6), GSE28829 (early vs. advanced plaques, *n* = 10), and GSE120521 (stable vs. unstable plaques, *n* = 3). Comparing gene expression levels, GLS remained significantly downregulated across the three datasets, and FDX1 and SLC31A1 were consistently upregulated in advanced and ruptured atherosclerotic plaques (Fig. 4d-e). This suggested that GLS may influence all stages of AS, while a higher level of FDX1 and SLC31A1 expression could predict more severe kinds of atherosclerotic plaques. Together, FDX1, GLS, and SLC31A1 were identified as the hub DE-CRGs in cuproptosis and AS.

### The diagnostic value of FDX1, GLS, and SLC31A1 in AS

**Fig. 5:**
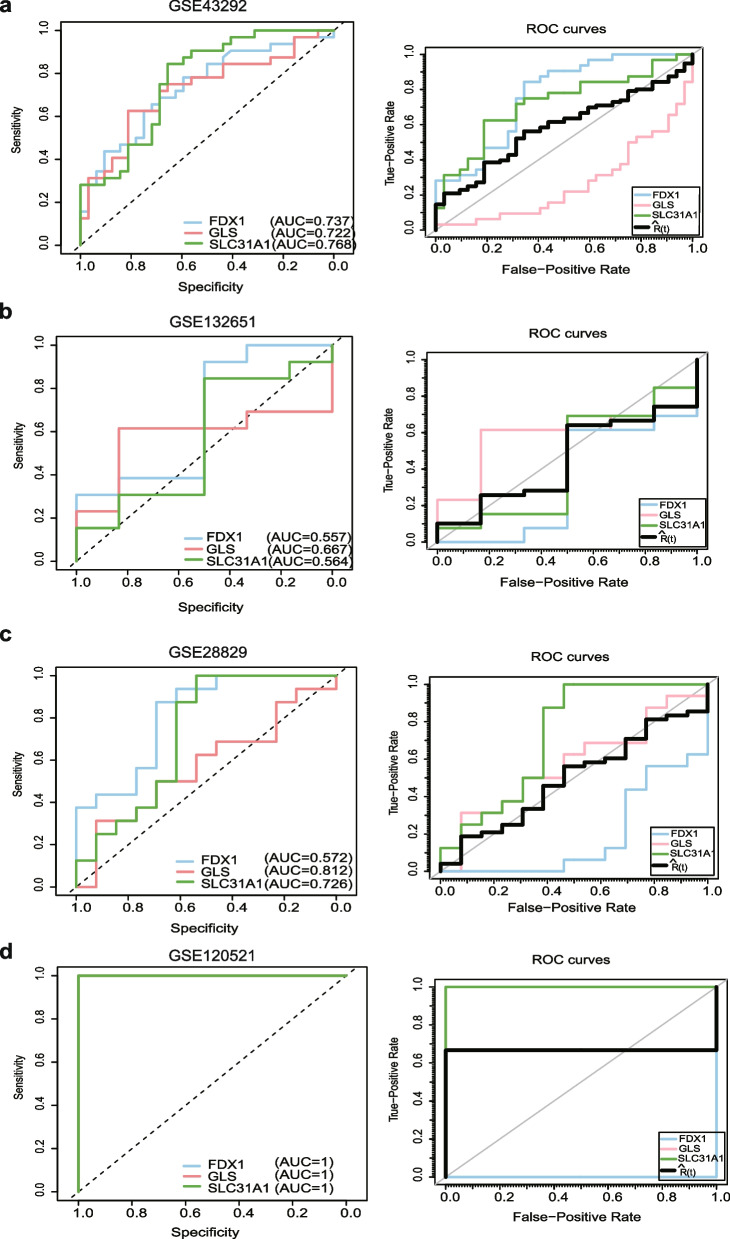
The diagnostic value of 3 Hub DE-CRGs in AS. **a**-**d** The diagnostic power of FDX1, GLS, and SLC31AL in atherosclerosis by ROC curve and intergroup overall inspection of ROC analysis in datasets GSE43292 (**a**), GSE132651 (**b**), GSE28829 (**c**), and GSE120521 (**d**)

ROC analysis and the area under the ROC curve (AUC) were performed in different datasets to evaluate the diagnostic value of three hub DE-CRGs in AS. ROC analysis demonstrated that FDX1, GLS, and SLC31A1 accurately predicted AS in GSE43292 (Fig. 5a). Furthermore, the overall inspection of the three ROC curves showed the same trend as the Student’s t-test. In GSE132561, GSE28829, and GSE120521, the ROC curves of FDX1, GLS, and SLC31A1 also verified that both had a high value for classification of the atherosclerosis stage (Fig. 5b-d). The AUCs for FDX1, SLC31A1, and GLS with the progression of atherosclerotic plaques showed significant performance prediction for samples (Fig. 5a-d). Thus, those hub genes had a potential diagnostic value for AS.

### Differential expression of FDX1, GLS, and SLC31A1 in human arterial tissues between atherosclerotic patients and healthy controls

**Fig. 6:**
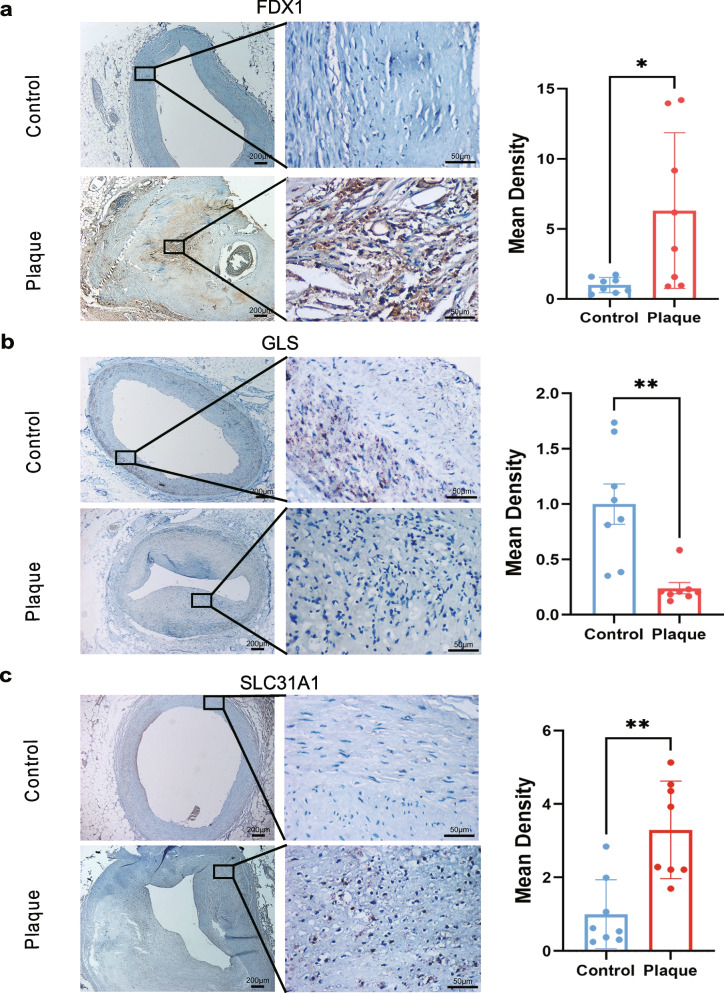
Immunohistochemistry for FDX1, GLS and SLC31A1. **a**-**c** Immunohistochemical staining of FDX1 (**a**), GLS (**b**), and SLC31A1 (**c**) in human normal coronary arterial tissues sections (Control) and AS tissues (Plaque), and mean density of FDX1 + (% area), GLS + (% area), SLC31A1 + (% area). Scale bar: 200 µm and 50 µm. ***P < 0.001, **P < 0.01 and *P < 0.05 vs. control group. *n* = 8

We examined FDX1, GLS, and SLC31A1 expression by immunohistochemical (IHC) staining. A total of 16 coronary artery sample sections were selected in each group, including eight from healthy subjects and eight atherosclerotic patients. Compared with the control group, FDX1 expression was increased (Fig. 6a, *P* < 0.01). The expression of GLS showed a significant decrease (Fig. 6b, *P* < 0.01). SLC31A1 showed upregulation in atherosclerotic plaques (Fig. 6c, *P* < 0.01). A negative control of IHC was presented in Supplementary Fig. 1A-C. In conclusion, the results of IHC were consistent with the analysis of the datasets, demonstrating that FDX1, GLS, and SLC31A1 were expressed significantly differently in AS plaques compared to normal tissues.

### Verification of cellular localization of FDX1, GLS, and SLC31A1 in atherosclerotic plaque

**Fig. 7:**
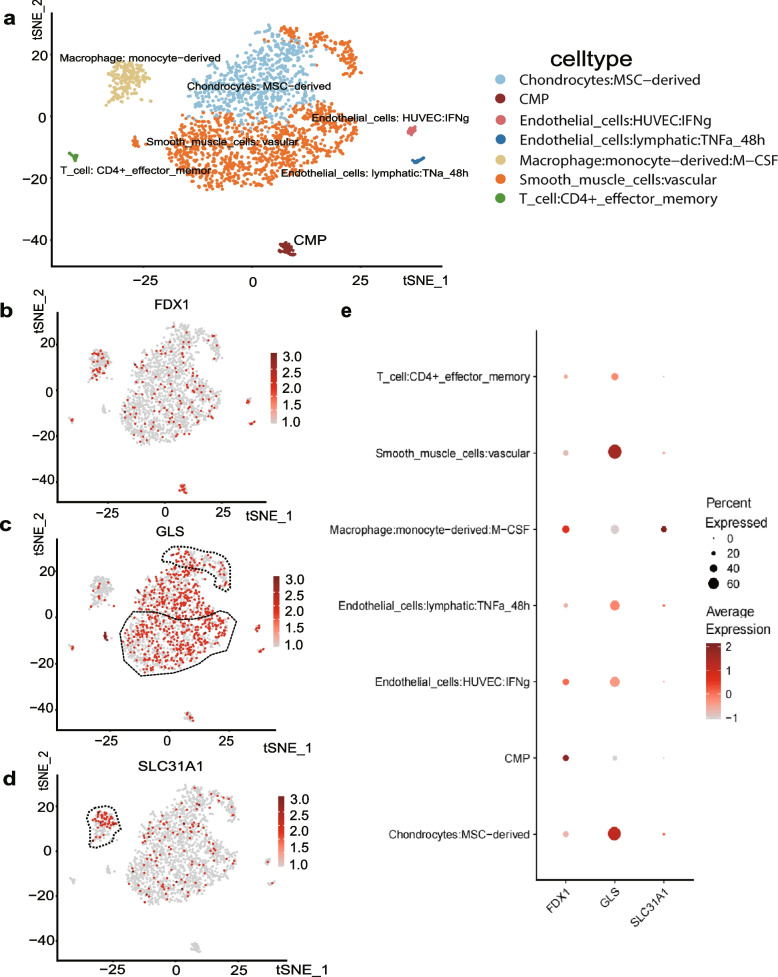
Cell localization and expression of FDX1, GLS, and SLC31A1 in AS single-cell data samples. **a** Classification of cell populations and location of specific genes in datasets GSE155514. **b**-**d** Dot plots (in red) showing the expression of three hub genes on the t-SNE map: FDX1 (**b**), GLS (**c**), and SLC31A1 (**d**). **e** Dot plot showing the expression of three hub genes in each cluster. Deeper and larger dots indicate higher expression levels

**Fig. 8:**
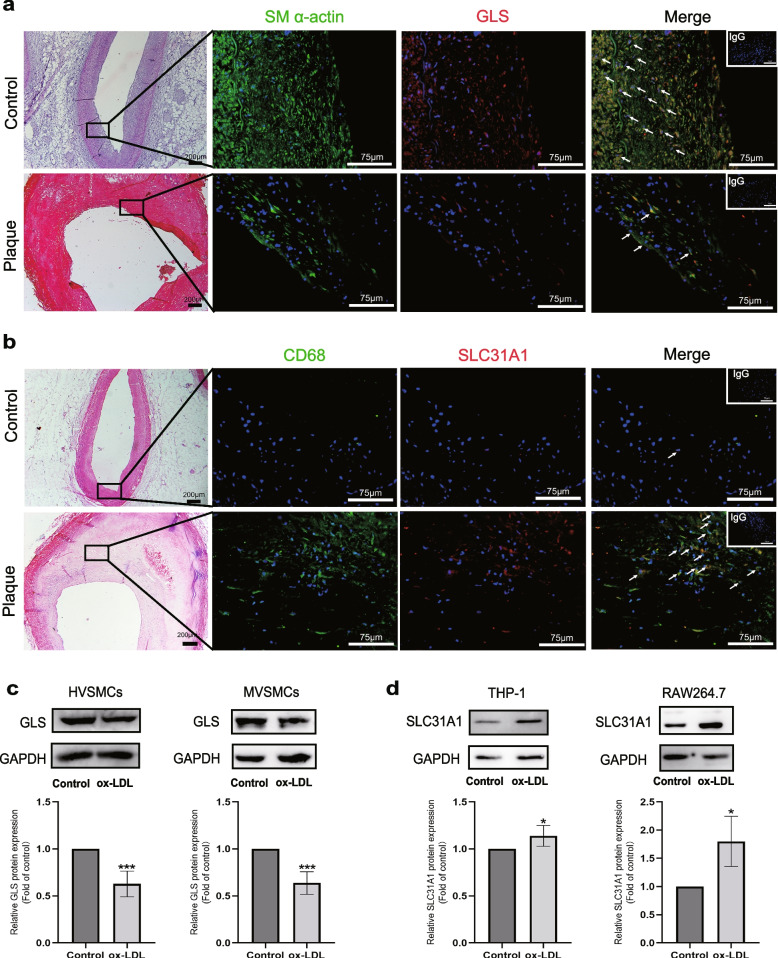
Verification of cellular localization of GLS, and SLC31A1 in atherosclerotic plaque. **a**-**b** Immunofluorescence staining of GLS (**a**) and SLC31A1 (**b**) in sections of normal human coronary arterial tissues (Control) and AS tissues (Plaque). GLS and SLC31A1 are shown in red, while SM α-actin and CD68 are shown in green. The black box indicates the 40 × fluorescence shooting area. White arrows point to colocalized cells. Scale bars are 200 µm and 75 µm. IgG was used as a negative control. **c**-**d** Western blot analysis of GLS protein expression in human vascular smooth muscle cells (HVSMCs) and mice primary vascular smooth muscle cells (MVSMCs), and SLC31A1 protein expression in THP-1 and RAW264.7 cells. The data were reported as mean ± SD (*n* = 4). ***P < 0.001, **P < 0.01 and *P < 0.05 vs. control group. *n* = 4

Identification of cell clusters in AS samples and tracking the cellular localization of screened hub genes proceeded as follows. First, we utilized the Seurat pipeline for single-cell analysis of AS sample in dataset GSE155512, which was performed on the carotid arteries of atherosclerotic patients. After data screening, Seurat object construction, quality control, dimensionality reduction, clustering, and cell annotation, the carotid intimal component of atherosclerotic patients were classified into seven large clusters of cells. We included vascular smooth muscle cells (VSMCs), mesenchymal stem cell-derived chondrocytes (MSCDCs), human umbilical vein endothelial cells (HUVECs), lymphatic endothelial cells, monocyte-derived macrophage cells, CD4 + T cells, and common myeloid progenitor (CMP) in the datasets (Fig. 7a). Next, we observed the clustering locations and average expression of the three hub genes on the T-SNE classification map and found that GLS was expressed in multiple cell clusters but concentrated in regions of VSMC distribution. SLC31A1 was more clearly expressed in the regions distributed by macrophages, while FDX1 was uniformly distributed in different atherosclerotic cell lines (Fig. 7b-e). Using double immunostaining in human plaques, we observed that the expression of smooth muscle α-actin (SM α-actin) in control and lesions was colocalized with GLS staining, and SLC31A1 also coincided with the fluorescence of macrophage localization marker CD68 (Fig. 8a-b). This result is consistent with the above bioinformatics inference.

For further support, we measured GLS and SLC31A1 protein expression in vitro. In line with these findings, compared to the control group, the expression of GLS was significantly decreased in the ox-LDL-treated human VSMCs (HVSMC) and mouse primary VSMCs (MVSMCs) (Fig. 8c). SLC31A1 had higher expression in the ox-LDL-treated human and mouse macrophages (Fig. 8d). These results indicate that FDX1, SLC31A1, and GLS are critical factors in AS, and cuproptosis may contribute to the development of AS.

## Discussion

Cuproptosis is defined as a nonapoptotic and copper-dependent cell death pathway. Copper can bind to lipoylated components of the TCA cycle, which leads to proteotoxic stress and cell death [16]. Copper plays an essential role in cardiovascular disease and has been suggested as an influence risk factor [17, 18]. Copper levels are known to be elevated in atherosclerotic lesions [3]. Indeed, copper was associated with an increase in the ten-year coronary risk and an increased risk of mortality from cardiovascular causes [17–19]. Copper deficiency can induce cardiovascular diseases, while copper excess can mediate various types of cell death, such as autophagy, apoptosis, cuproptosis, pyroptosis, and cardiac hypertrophy and fibrosis. Clarifying the correlation between cuproptosis and AS may provide novel biomarkers and ideas for diagnosis and therapy.

FDX1, an iron-sulfur protein, participates in a wider spectrum of metabolic processes by the TCA cycle, including iron-sulfur cluster biogenesis, bile acid synthesis, vitamin D synthesis, and steroidogenesis [20, 21]. Recent studies point out that FDX1 is the key regulator in cuproptosis, which can directly bind to copper and activate the proteotoxic stress [17, 18, 20–25]. Indeed, genome-wide association studies identified FDX1 as a susceptibility loci in the coronary artery disease [26]. Our results show it is similarly elevated in atherosclerotic lesions and has potential diagnostic value for AS. Single-cell sequencing suggested that FDX1 has a widespread distribution in different atherosclerotic cell populations. These results indicate that FDX1 may be one of the critical target genes that regulate cuproptosis in AS.

One of the crucial mechanisms of cuproptosis is intracellular copper accumulation. The copper transporters SLC31A1 to input copper from circulation and maintain copper homeostasis of most cells [27]. Nadina Stadler et al. detected elevated levels of total copper in carotid lesions [3]. Here, we detected upregulated SLC31A1 in human atherosclerotic plaques. These results may suggest that excess copper in the plaque was mediated by SLC31A1. Extensive neovascularization is a classical histological feature of an unstable plaque [28, 29]. Das et al. demonstrated that SLC31A1 could sense reactive oxygen species (ROS) to induce angiogenesis [30]. In AS, macrophage accumulation is directly linked to the destabilization and rupture of plaque, causing acute atherothrombotic events [31]. Our study shows that SLC31A1 was specifically upregulated in advanced plaque or unstable plaque, which are rich in macrophages. Furthermore, we also found ox-LDL-induced SLC31A1 upregulation in macrophages. Taken together, it would be reasonable to believe that SLC31A1 could induce macrophage cuproptosis to promote plaque instability.

GLS is the enzyme that catabolizes glutamine to glutamate and ammonia. GLS-driven catabolism of glutamine serves not only for energy production but also for the maintenance of the redox state. GLS breaks down glutamate production of glutamine, which in turn leads to increased synthesis of intracellular glutathione (GSH) to resist ROS-induced damage and protect cells from copper toxicity [32–35]. Indeed, the current study showed the downregulation of GLS transcripts in human plaque tissue compared with normal vessels, and double-fluorescence labeling confirmed that GLS was localized in VSMCs. These results suggest that excessive copper destroys the GLS-driven oxidation–reduction system of VSMCs, which aggravates the toxic damage of copper, thus promoting AS.

In summary, we have first identified three CRGs, FDX1, SLC31A1, and GLS, in the development of AS. Our results also suggest FDX1, SLC31A1, and GLS as potential diagnostic biomarkers for AS, providing more insights into the vital role of cuproptosis in AS. However, the studies on cuproptosis and AS are still in their infancy. More extensive studies are still needed to determine the detailed molecular mechanism of cuproptosis, thus providing more evidence for cuproptosis in the prevention and treatment of AS.

## Materials and methods

### Data collection and acquisition of cuproptosis-related genes (CRGs)

The bulk sequencing data and single-cell transcriptome data were collected from the GEO database with series numbers GSE97210, GSE43292, GSE120521, GSE 132,651, GSE28829, and GSE155512. Patient information from the GEO datasets used is available on the GEO website (https://www.ncbi.nlm.nih.gov/geo/). A total of 16 CRGs, retrieved from the published articles, were obtained for subsequent analyses [16].

### Identification of differentially expressed CRGs (DE-DRGs)

The “limma” and “edgeR” packageswere used to calibrate the microarray data and identify the differentially expressed genes (DEGs) between the atherosclerotic plaques and normal arterial intimae in GSE97210 datasets [36, 37], which characterized the mRNA profiles in advanced AS or normal intima tissues. The mRNAs that met the defined criteria were considered as DEGs (adj.pval < 0.05, |logFC|> 1). Then, the intersecting genes between DEGs were defined as the DE-CRGs. By using the OmicStudio tools at https://www.omicstudio.cn/tool [38], the DEGs were displayed in volcano plots, and the DE-CRGs were visualized in a heatmap. The number of CRGs was shown in a Venn diagram. Correlation analysis of the differential expression of CRGs in GSE97210 datasets was based on the “corrplot” package [39].

### Functional annotation, pathway enrichment, and construction of Protein–Protein Interaction (PPI) network

Gene Ontology (GO) biological process and Kyoto Encyclopedia of Genes and Genomes (KEGG) annotation were performed by the DAVID website [40], and the “ggplot2” package was used to render the results of the GO biological process and KEGG pathway in the bar plots. The STRING database was employed to analyze the interactions of the DE-CRGs and then used to construct and visualize the PPI network.

### Screening cuproptosis-related candidate biomarkers

The expression levels of DE-CRGs in the control group and AS group were analyzed by Student’s t-test in datasets GSE97210 and GSE43292, with a *P* value less than 0.001 considered to be significant. To evaluate the diagnostic value of DE-CRGs for AS, a Random Forest (RF) classification model was implemented with the package “randomForest”. Thereafter, three Hub genes with significant expression of the same trend in the two datasets and with high confidence scores in the RF model were selected for further analysis. Receiver operating characteristic (ROC) curves of three Hub genes were plotted, and areas under the ROC curve (AUC) were calculated using the “pROC” package [41]. The three microarray datasets of atherosclerotic plaque (GSE132651: normal vs. atherosclerotic endothelial; GSE28829: early vs. advanced plaques; and GSE120521: stable vs. unstable plaques) were then used to verify the expressions and diagnostic value of the Hub genes.

### Cluster analysis for single-cell transcriptomic datasets

The data matrix of sample GSM4705589 from GSE155512 datasets was transformed into a “Seurat object” using the package “Seurat” [42]. The quality was controlled by sequencing gene count and expression and mitochondrial gene percentage. The "NormalizeData" function was applied to standardize the expression matrix, and the "RunPCA" function was used for PCA dimensionality reduction analysis. Inputting the first three principal components, we subsequently performed density clustering to identify the composition of vascular endothelial and atherosclerotic plaques and visualized it through t-distributed statistical neighbor embedding (t-SNE). Moreover, the package of “SingleR” was used for the cell-type annotation [43].

### Histological, immunohistochemical, and immunofluorescence analyses of arterial tissues

Autopsy specimens were collected from accidental death cases and from individuals with varying degrees of AS lesions in the aorta, and were obtained from the University of South China Forensic Identification Center. Additional details are provided in Supplementary Table 1. The research protocol was approved by the Medical Ethics Committee of the University of South China (Approval number: USC202209HT17). Tissue samples were embedded in paraffin blocks for histology studies. For immunofluorescence, after deparaffinization and dehydration, endogenous peroxidase was blocked with 3% hydrogen peroxide, and non-specific binding was blocked with 10% fetal bovine serum (FBS). Primary antibody was added for incubation overnight at 4℃. The sections were incubated with polymerized horseradish peroxidase (HRP)-anti-rabbit IgG and visualized using DAB. The integrated optical density (IOD) and area of five fields were randomly selected from each sample to calculate the mean density, which was used as the final value of each sample. For co-localization of immunofluorescence, after deparaffinization and dehydration, the sections were blocked with 10% FBS, followed by incubation with primary and secondary antibodies to target gene and marker proteins. The nuclei were counterstained with 4',6-Diamidino-2-phenylindole (DAPI), followed by fluorescence microscopy. The same concentration of isotype control IgG was used as a negative control. Antibodies against FDX1(ZEN GIO, RRID: R23394), GLS (HUABIO, ET1611-5), SLC31A1 (Bioss, bs-100773R), α-SMA (Proteintech, 67,735–1-gl), and CD68 (Abcam, ab201340) were used.

### Cell culture conditions

Human aortic SMCs (CRL-1999) were purchased from BeNa Culture Collection (BNCC, Beijing, China) and authenticated by the supplier. Human THP-1 monocytes were purchased from ATCC. RAW264.7 macrophages were obtained from the Chinese Academy of Sciences. Mouse primary vascular smooth muscle cells (VSMCs) were extracted from freshly dissected thoracic aortic segments of male four- to six-week-old C57/B6 mice: First, samples of thoracic aortic segments isolated from mice were initially digested in enzyme mixtures (Worthington, LS003570, LS004174, LS00227). Then, the adventitia and intima were removed, leaving only the media portion of the vessel. Finally, the remaining vascular tissue was cut up and digested again with mixed enzymes to isolate primary mouse VSMCs. Both human and mouse VSMCs were cultured in DMEM/F-12 medium (Gibco, C11330500BT) containing 10% FBS (Gibco) and 1% penicillin/streptomycin (Biosharp, Hefei, China). at 37℃ with 5% CO_2_. Cells were starved for 8 h and then treated with oxidized low-density lipoprotein (ox-LDL) (50 mg/ml) for 24 h.

### Western blot

Protein expression was detected by Western blotting. Proteins extracted from cells were processed with RIPA lysis buffer, and concentrations were measured using the BCA Protein Assay Kit (Thermo Scientific, 23,227). After ultracentrifugation at 4℃, the supernatant was added to the loading buffer and loaded onto 8% SDS-PAGE gels for electrophoresis and transfer topolyvinylidene difluoride (PVDF) membranes. The membranes were blocked with 5% skim milk, incubated with primary antibody (overnight at 4℃), then washed three times in Tris Buffered Saline with Tween (TBST), and incubated with secondary antibody (2 h at room temperature). The bands were visualized by Enhanced Chemiluminescence (ECL) substrate. Antibodies against GLS (HUABIO, ET1611-5), SLC31A1 (HUABIO, HA720005), and GAPDH (Proteintech, 10,494–1-AP) were used.

### Statistical analysis

All obtained images were submitted to Image-Pro Plus software (version 6.0) and Image J software for quantification, and statistical analyses were performed using GraphPad Prism software (version 9.0). All bioinformatics analyses used R software (version 4.2.0). The Shapiro–Wilk normality test was used to analyze the normality of the data distribution. Descriptive data are expressed as mean ± SD. The differences between the two groups were compared with a Student's t-test, and a one-way analysis of variance (ANOVA) was performed to compare multiple groups. Pearson correlation analysis was used for normally distributed variables, and Spearman correlation analysis was used for non-normally distributed variables. Results were considered statistically significant when *P* values were less than 0.05.

## Supplementary Information

**Additional file 1: Fig. 1.** Negative control of Immunohistochemistry. A-C Immunohistochemical staining and corresponding negative control for FDX1 (A), GLS (B), and SLC31A1 (C) in human coronary arterial tissue sections.

**Additional file 2: Table 1.** Patient details. Arterial plaques were graded according to the Oxford grading system. All human arterial samples were obtained with informed consent, and the procedures were performed in accordance with institutional guidelines.

## Data Availability

All data presented are contained within the manuscript.

## Abbreviations

AS: Atherosclerosis
α-SMA: α- Smooth muscle actin
CVD: Cardiovascular disease
CRGs: Cuproptosis-related genes
DEGs: Differentially expressed genes
DE-CRGs: Differential cuproptosis-related genes
FDX1: Ferredoxin 1
GLS: Glutaminase
GO: Gene Ontology
KEGG: Kyoto Encyclopedia of Genes and Genomes
LDL: Lipoprotein
ox-LDL: Oxidized low-density lipoprotein
RF: Random Forest
ROC: Receiver operating characteristic
SLC31A1: Solute carrier family 31 member 1
TCA: Tricarboxylic acid
T-SNE: T-distributed statistical neighbor embedding
VSMCs: Vascular smooth muscle cells
