# Supplementary figures and images for: A novel cuproptosis-related diagnostic gene signature and differential expression validation in atherosclerosis
Source: Mol Biomed. 2023 Jul 14;4:21. doi: 10.1186/s43556-023-00131-5 (PMC10344858; doi:10.1186/s43556-023-00131-5)

**A**

FDX1

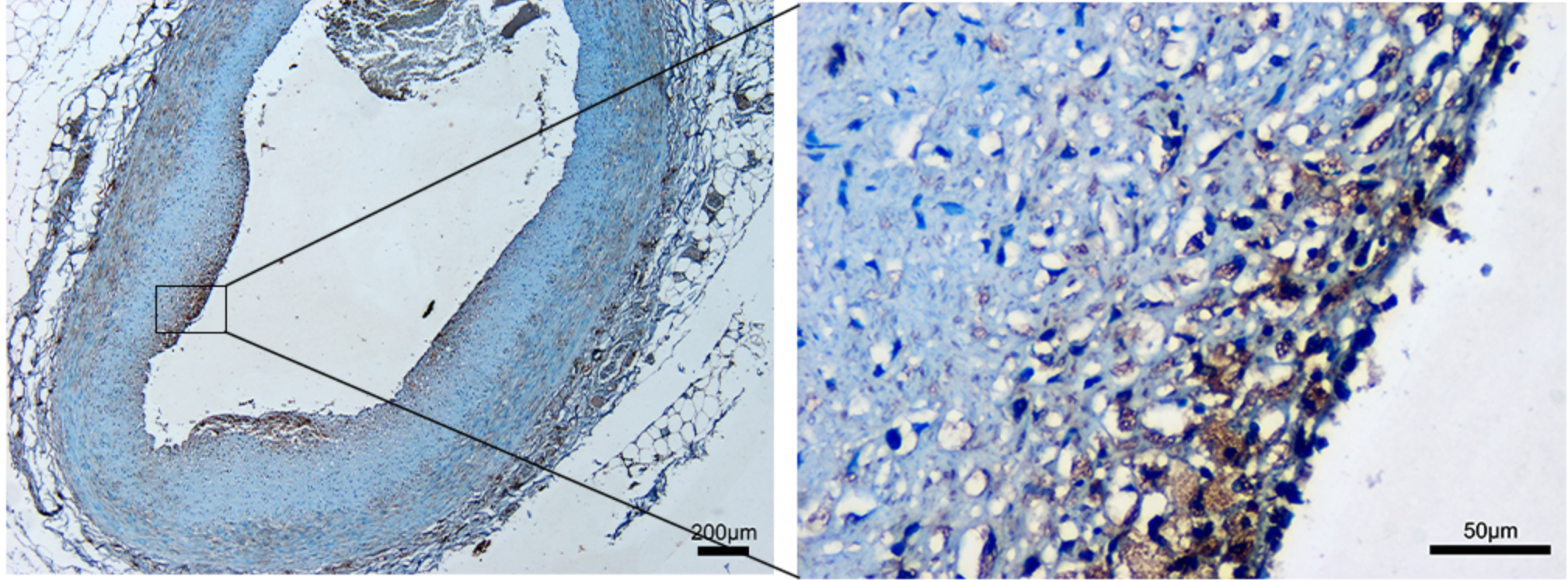

Negative control

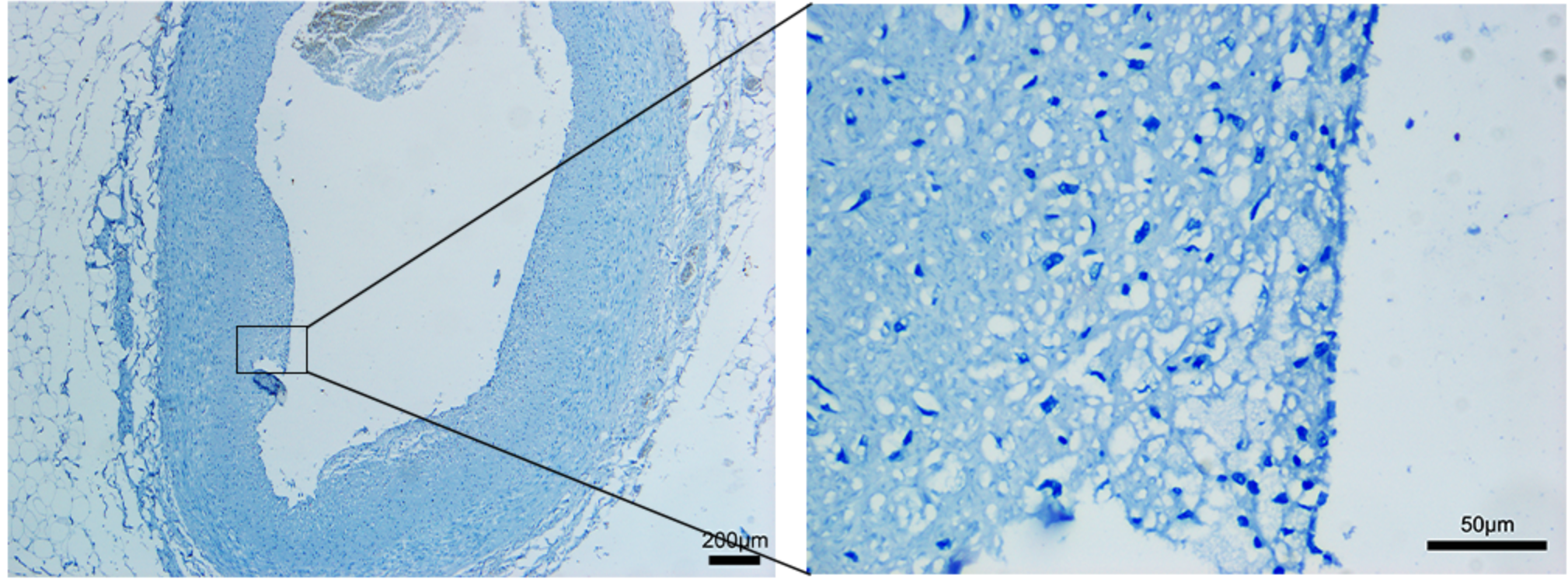

**B**

GLS

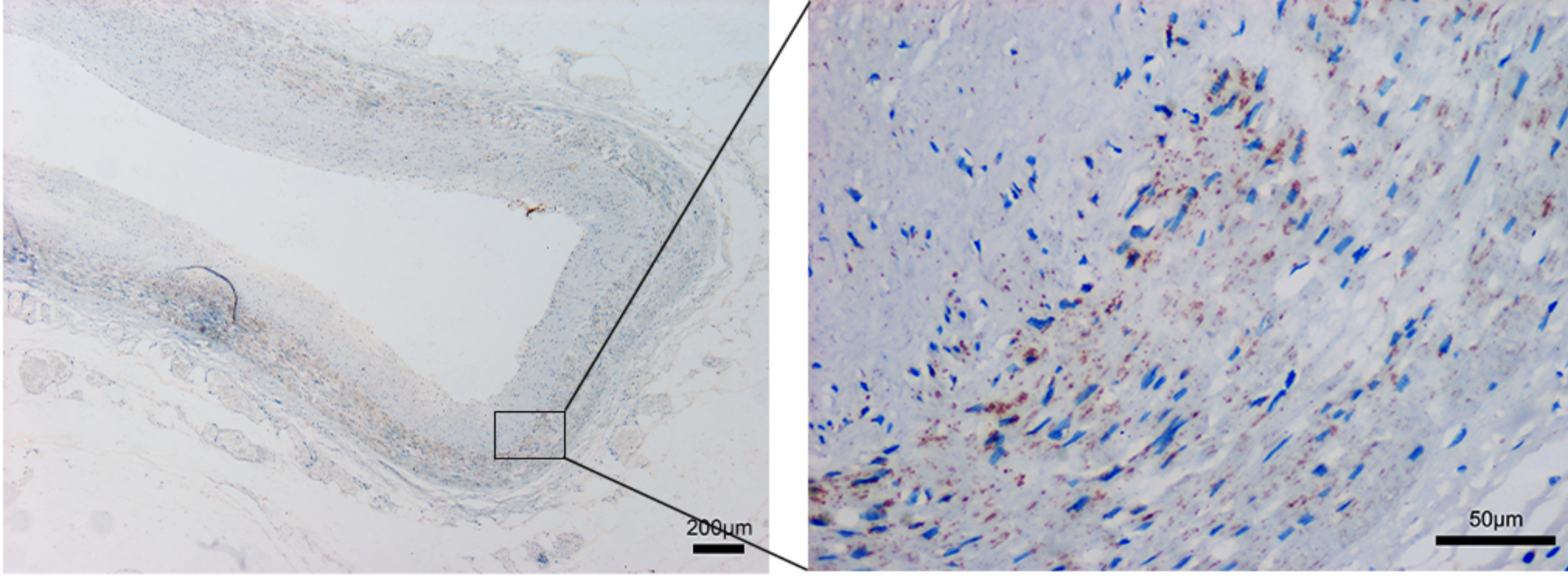

Negative control

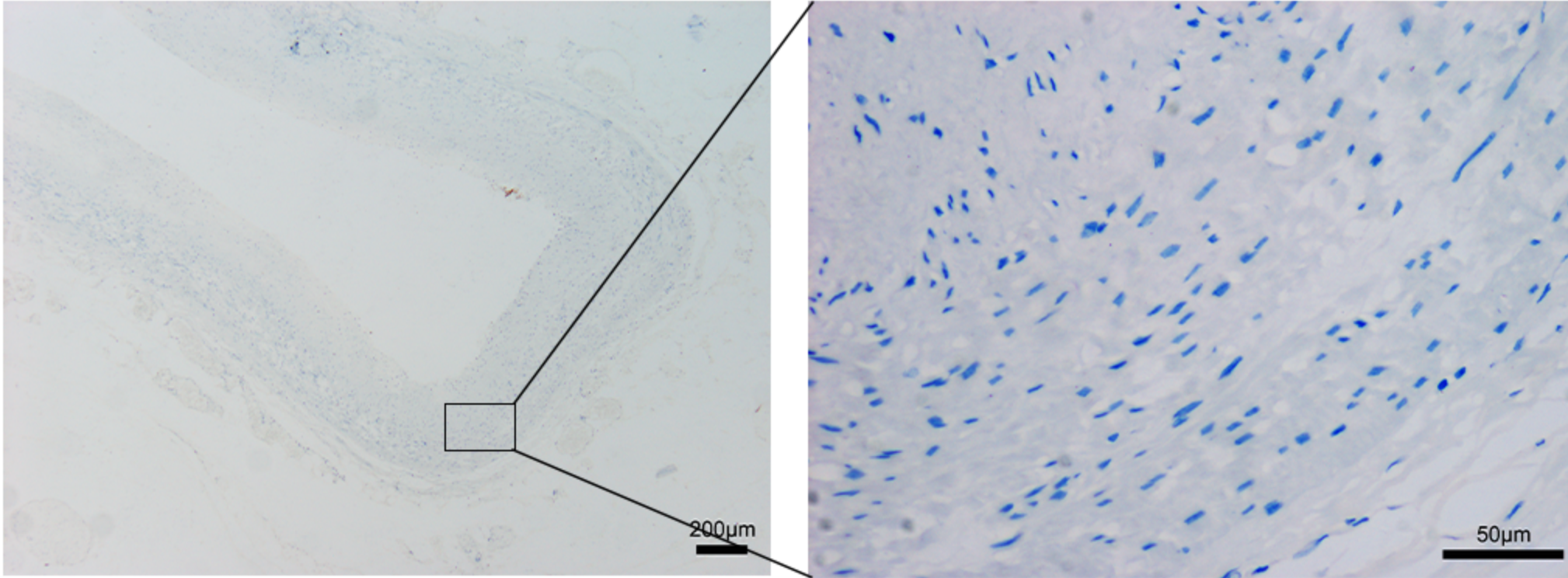

**C**

SLC31A1

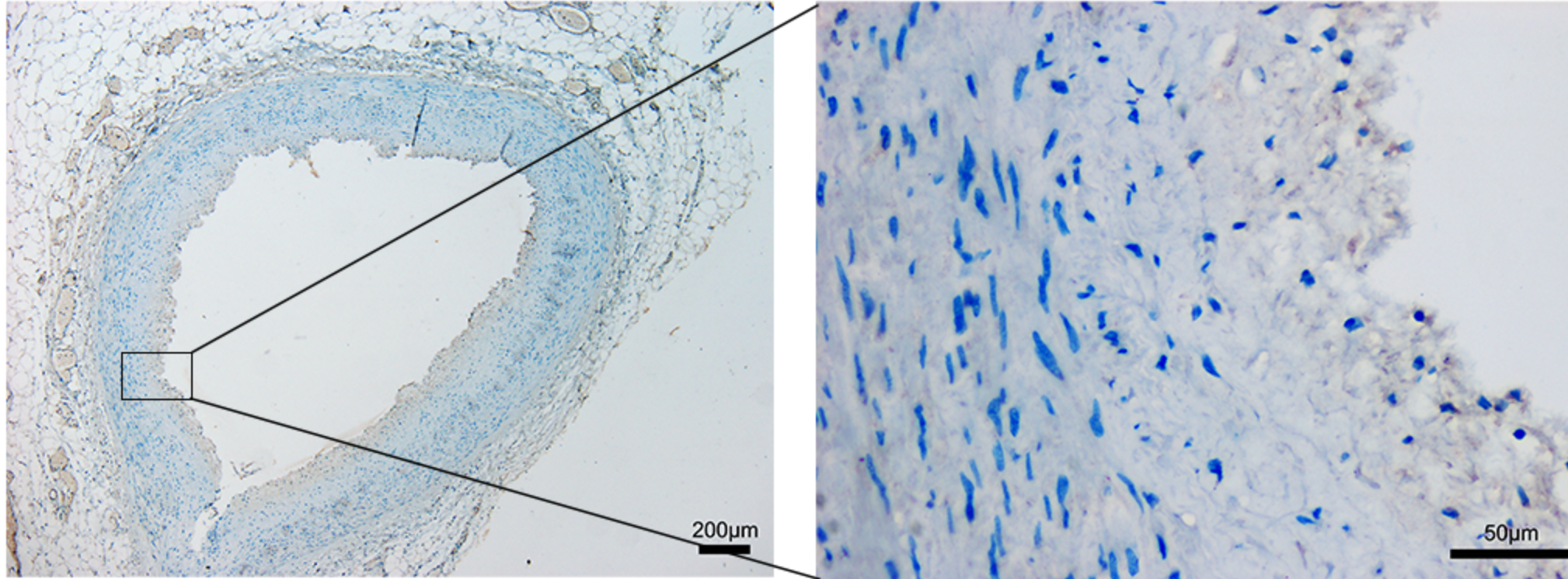

Negative control

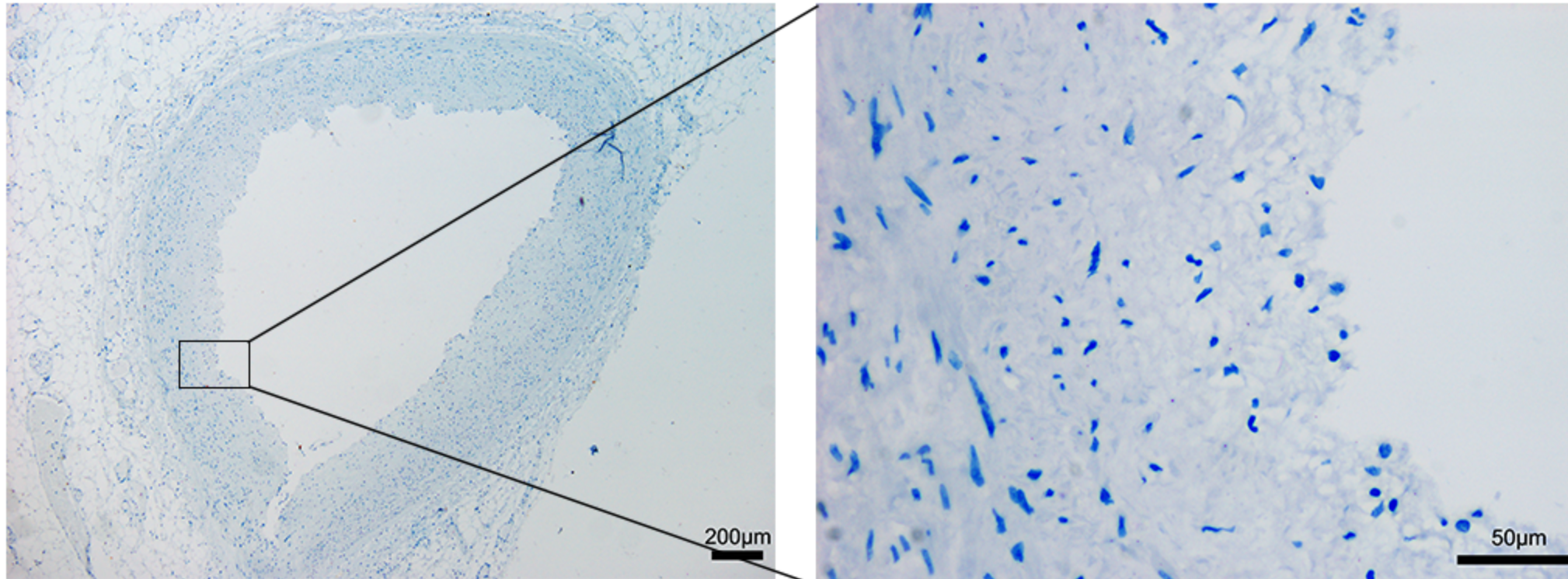

Supplement: Supplementary file 1 — Additional file 1: Fig. 1. Negative control of Immunohistochemistry. A-C Immunohistochemical staining and corresponding negative control for FDX1 (A), GLS (B), and SLC31A1 (C) in human coronary arterial tissue sections. [file 43556_2023_131_MOESM1_ESM.pdf]
