# Supplementary material for: A novel cuproptosis-related diagnostic gene signature and differential expression validation in atherosclerosis
Source: Mol Biomed. 2023 Jul 14;4:21. doi: 10.1186/s43556-023-00131-5 (PMC10344858; doi:10.1186/s43556-023-00131-5)
Supplement: Supplementary file 2 — Additional file 2: Table 1. Patient details. Arterial plaques were graded according to the Oxford grading system. All human arterial samples were obtained with informed consent, and the procedures were performed in accordance with institutional guidelines. [file 43556_2023_131_MOESM2_ESM.pdf]

Supplementary Table 1 Patient details

Serial number	Gender	Age	Histopathological grade of plaque	Ethnicity
1	Female	47	NA	Asian
2	Female	22	NA	Asian
3	Male	37	NA	Asian
4	Female	32	NA	Asian
5	Male	19	NA	Asian
6	Male	46	NA	Asian
7	Female	48	NA	Asian
8	Female	37	NA	Asian
9	Male	30	NA	Asian
10	Female	20	NA	Asian
11	Male	79	II	Asian
12	Male	64	IV	Asian
13	Male	51	I	Asian
14	Male	40	I	Asian
15	Male	63	III	Asian
16	Male	81	III	Asian
17	Male	47	IV	Asian
18	Male	48	I	Asian
19	Male	54	III	Asian
20	Male	38	III	Asian
21	Female	76	III	Asian
22	Male	61	III	Asian
23	Female	55	II	Asian
